# Flowers for the London Poor

**Published:** 1887-07-02

**Authors:** T. E. Tripp

**Affiliations:** Hon. Sec. Flower Distribution Branch of the Kyrle Society. The Kyrle Society, 14, Nottingham Place, W.


### FLOWERS FOR THE LONDON POOR.

Sir,—Although the backward spring has this year diminished the supply of flowers, I venture to hope that there may still be some available for distribution among the poor of London. I trust that it is hardly necessary for me to enlarge upon the pleasure with which the flowers are received by the poor, and I will only say that I shall still hopefully look for offers of both plants and flowers. A special demand is made for plants in pots, which can be used for the wards of hospitals and workhouses.

In conclusion, I will ask all those who are kind enough to promise contributions not to send them to the office, but to communicate first with me, when I will give addresses to which they may be sent direct. I shall also be much obliged if donors will tell me how often they can send flowers, and if they wish the hampers returned.

T. E. Tripp,
Hon. Sec. Flower Distribution Branch
of the Kyrle Society.

The Kyrle Society, 14, Nottingham Place, W.

